# Clinical and Prognostic Analysis of Autoantibody-Associated CNS Demyelinating Disorders in Children in Southwest China

**DOI:** 10.3389/fneur.2021.642664

**Published:** 2021-03-26

**Authors:** Ziyan Li, Hong Sun, Xiao Fan, Ping Yuan, Yan Jiang, Peng Wu, Min Zhong, Jiannan Ma, Li Jiang, Xiujuan Li

**Affiliations:** ^1^Department of Neurology, Children's Hospital of Chongqing Medical University, Chongqing, China; ^2^Chongqing Key Laboratory of Pediatrics, Ministry of Education Key Laboratory of Child Development and Disorders, National Clinical Research Center for Child Health and Disorders, China International Science and Technology Cooperation Base of Child Development and Critical Disorders, Children's Hospital of Chongqing Medical University, Chongqing, China; ^3^Department of Radiology, Children's Hospital of Chongqing Medical University, Chongqing, China

**Keywords:** children, myelin oligodendrocyte glycoprotein (MOG), aquaporin-4 (AQP4), acquired demyelinating syndromes (ADS), serostatus

## Abstract

**Objective:** To analyze the positive and recurrence rates of different autoantibody-associated demyelination disorders in children in Southwest China, and describe the clinical, radiological, and prognostic features of the myelin oligodendrocyte glycoprotein antibody (MOG-ab) and aquaporin-4 antibody (AQP4-ab) associated disease. This study also summarizes steroid maintenance therapy approaches for MOG-ab-positive children.

**Methods:** A total of 160 children presenting with acquired demyelinating syndromes (ADS) between January 2016 and December 2019 were tested for MOG-ab and AQP4-ab. Clinical data, MRI scans, and survival analyses were compared between MOG-ab-positive and AQP4-ab-positive children. Evolution of serologic status and treatment response to immunosuppressants were collected in MOG-ab-positive children.

**Results:** Of the 160 included children, the MOG-ab positivity rate (47.4%) was significantly higher than the AQP4-ab (5%) positivity rate. The recurrence rate for AQP4-ab disease (71.4%) was higher than that of MOG-ab disease (30.1%). For 135 children with both MOG-ab and AQP4-ab tested, the median age at onset was 7 (interquartile range [IQR] 5–10) years, and the median follow-up period was 19 (IQR 13–27.5) months. MOG-ab-positive children more frequently presented with acute disseminated encephalomyelitis, had deep gray matter lesions on MRI, had a better clinical and radiological recovery, and were less likely to have sustained disability than AQP4-ab-positive children. In MOG-ab-positive and AQP4-ab-positive children, maintenance therapy was a protective factor for recurrence, but presenting optic neuritis was a predictor of earlier relapse. A high Expanded Disability Status Scale score at onset was associated with sustained disability. Steroid maintenance therapy longer than 6 months after the initial attack was associated with a lower risk of a second relapse in MOG-ab-positive children. On serial serum MOG antibody analysis, clinical relapse occurred in 34.6% of children with persistent seropositivity, but none of the children who converted to seronegative status experienced relapse.

**Conclusion:** The MOG antibody is more common in children with ADS than the AQP4 antibody. MOG-ab-positive children are characterized by distinct clinical and radiological features. Although some MOG-ab-positive children experience relapsing courses or have persistently seropositive status, they still predict a better outcome than AQP4-ab-positive children.

## Introduction

Acquired demyelinating syndromes (ADS) are a group of inflammatory diseases characterized by acute-onset neurological deficits associated with evidence of central nervous system (CNS) demyelination (1). Presentations include optic neuritis (ON), transverse myelitis (TM), encephalopathy, or other syndromes attributed to brainstem/cerebellar or hemisphere involvement. ADS may occur as a monophasic illness or herald the onset of chronic relapsing disorders. In the spectrum of ADS, a substantial overlap in clinicoradiological presentations exists in a variety of these disorders, making it difficult to distinguish each other in individuals (2). Apart from the incorporation of clinical and neuroradiological features in diagnostic work-up, specific biological markers and new-generation antibody assays have expanded the knowledge and definitions of unique disease entities. An elevated immunoglobulin index or the presence of oligoclonal bands (OCB) in the cerebrospinal fluid (CSF) has helped diagnose multiple sclerosis (MS) (2), and the discovery of pathogenic serum autoantibodies target against aquaporin-4 (AQP4-abs) plays an important role in the novel classification of neuromyelitis optica spectrum disorders (NMOSD) (3).

Myelin oligodendrocyte glycoprotein (MOG) is a glycoprotein expressed on the outermost surface of myelin sheaths and oligodendrocyte membranes. Although its biological role remains elusive, several studies have demonstrated its utility as a target to differentiate inflammatory demyelinating diseases (IDDs) and its roles as a cellular receptor, an adhesion molecule, and a regulator of microtubule stability (4, 5). MOG-antibody-associated disorders (MOGAD) are identified as entities with either different nosological, treatment response, or prognostic parameters (6–8), and no extant IDD covering all its manifestations according to current criteria or terminology. In recent years, numerous studies have found that the MOG antibody (MOG-ab) was associated with some rare and atypical demyelination types, which may expand the spectrum of MOGAD, such as unilateral cerebral cortical encephalitis (9) and meningitis (10).

In this study, we retrospectively investigated children with ADS at a single center to evaluate frequency and relapse rates according to antibody serostatuses. We then focused on MOG-ab-positive children to describe the clinical, radiological, and prognostic features and assess the value of serum longitudinal analyses of MOG-ab titers during the course of the disease in each patient. We also hope to develop an oral prednisone taper program as a reference, based on our empirical treatment of MOG-ab-positive children.

## Methods

### Participants

**Figure 1:**
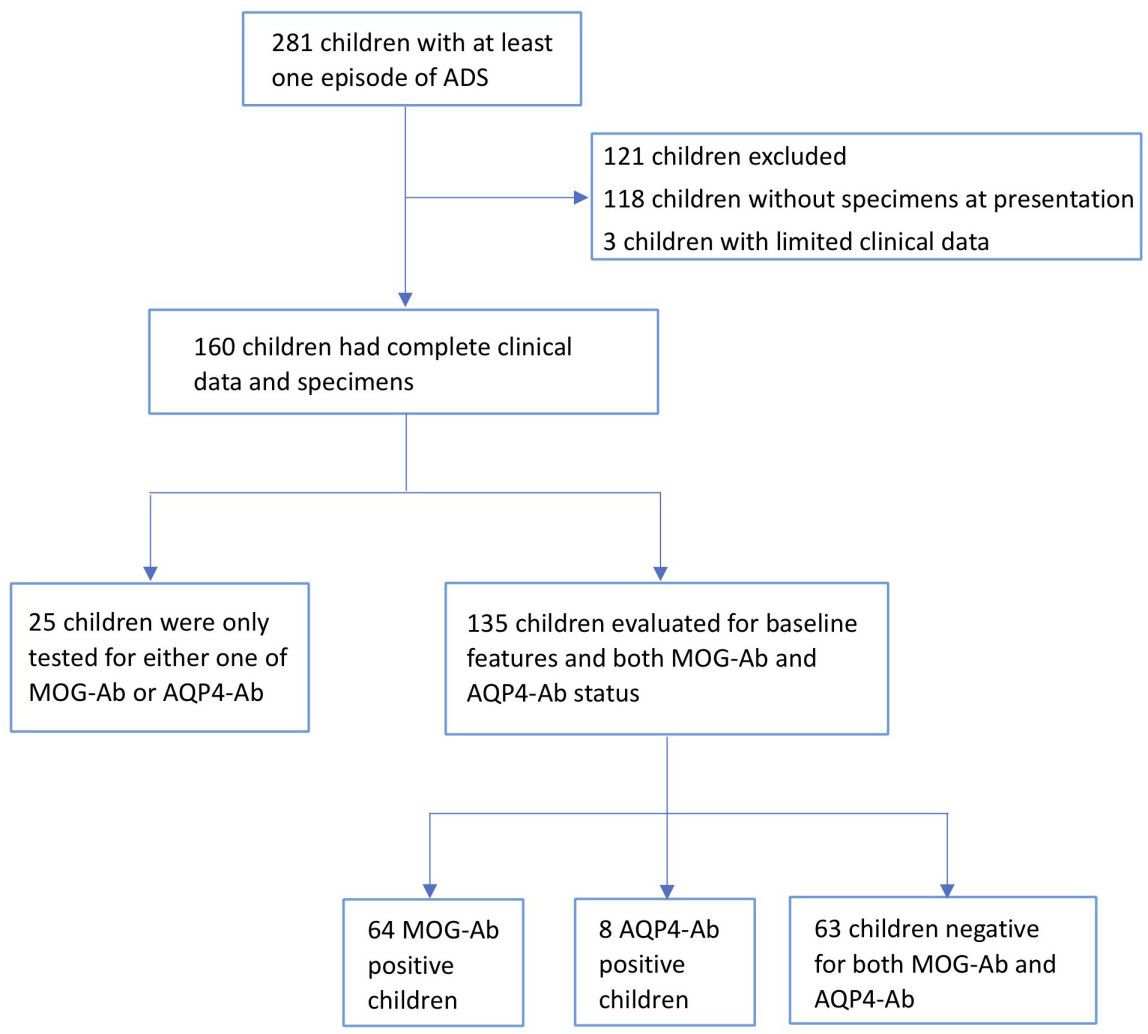
Flow diagram. A total of 160 participants with acquired demyelinating syndromes (ADS) with complete clinical data and serum or cerebrospinal fluid (CSF) specimens were tested for MOG-antibody (MOG-ab) and AQP4-antibody (AQP4-ab). For 135 participants with both antibodies tested, 64 participants were MOG-ab-positive, 8 participants were AQP4-ab-positive, and 63 participants were negative for both antibodies.

We retrospectively observed 281 children presenting with at least one acute clinical episode consistent with an ADS of the CNS between January 2016 and December 2019 at the Children's Hospital of Chongqing Medical University. Exclusion criteria included the presence of infectious, metabolic, vascular, or neoplastic central nervous system disease. Of these, 160 participants who provided complete clinical data and serum, or CSF specimens within three months of onset or at the time of clinical relapse, were included in the study (Figure 1).

### Procedure

We reviewed all clinical records of the eligible participants and summarized the positivity and relapse rates of the 160 patients with AQP4-antibody (AQP4-ab) and MOG-ab, identified in their specimens. Of these, 135 patients were evaluated for both antibodies and were divided into MOG-ab-positive group, AQP4-ab-positive group, and both antibodies negative group.

Clinical phenotypes at onset included ON, myelitis, and acute disseminated encephalomyelitis (ADEM) who fulfilled established criteria (11), while encephalitis other than ADEM was defined as patients with criteria of encephalitis (12) who did not fulfill the criteria for ADEM. The final diagnoses were assigned as follows: MS, clinically isolated syndrome (CIS), ADEM (11), and NMOSD (13). Patients who were unclassifiable according to current criteria or terminology at the last follow-up were grouped into uncategorized syndromes.

Relapses were defined as the development of new neurological symptoms 1 month after the onset of the incident illness or, in the case of ADEM, 3 months after the onset of the initial episode (11). Clinical outcomes were subdivided into normal (no neurological symptoms), minor residuals (mild neurological symptoms, e.g., bladder dysfunction), or severe residuals (persistent significant neurological symptoms or sequelae such as epilepsy or learning difficulties) (14). Disability outcome was recorded using extrapolated Expanded Disability Status Scale (EDSS) scores. Sustained disability was defined as an EDSS score ≥ 2.0, persisting for at least 6 months.

Maintenance corticosteroids were defined as oral corticosteroids at daily doses of <2 mg/kg. Other immunosuppressive agents included rituximab (RTX), mycophenolate mofetil (MMF), and azathioprine (AZA). We calculated the time and annualized relapse rates (ARRs) before and after each maintenance therapy.

Clinical follow-up information including neurological symptoms and therapeutic response were obtained subsequently by the referring physicians or via a standard questionnaire in most cases in at least 6 months from the onset episode. One MOG-ab-positive patient and one AQP4-ab-positive patient were lost to follow-up.

### Autoantibody Detection and Cerebrospinal Fluid Studies

MOG-ab was detected by cell-based assay (CBA) using an in-house method. HEK293T cells were transfected with a pIRES2-EGFP-hMOG plasmid for 36 h. Transfected cells were incubated with patients' serum diluted at 1:10. After removing the media and washing with PBS, HEK293T cells were fixed with 4% PFA for 20 min and blocked with 5% goat serum for 30 min. Cells were then immunolabeled with an AlexaFluor 546 secondary antibody against human IgG (1:1000; Thermo Scientific) for 1 h at room temperature. AQP4-ab was detected with a CBA method using HEK293T cells transiently co-transfected with pcDNA3.1-EGFP-hAQP4. Two independent masked assessors evaluated each sample based on the intensity of surface immunofluorescence. MOG-ab and AQP4-ab titer levels of ≥1:10 were classified as seropositive. Titration of positive samples was performed using serial dilution until loss of positive signal (from 1:100 to 1:1000). Serial serum longitudinal analyses of MOG-ab titers were performed in 42 patients at intervals of more than three months. In particular, the significance of antibody positive and diagnoses of these patients was closely combined with clinical features and imaging data.

The presence of OCB or immunoglobulin G (IgG) index in CSF were assessed with isoelectric focusing on agarose gels followed by laboratory immunoblotting.

### Blinded Radiologic Analysis

MRI studies were analyzed to determine lesion locations separately by two neuroradiologists who were unaware of the patients' clinical data and Ab statuses. The MRI exams were performed with different scanners, either 1.5T or 3.0T magnetic strength according to the following parameters: T1-weighted, T2-weighted, fluid-attenuated inversion recovery (FLAIR), and T1-weighted postcontrast sequences. Scans were scored using a revised version of a published MRI scoring tool (15). The majority of patients were scheduled for brain or spinal imaging within 3 months of symptom onset; some had optic imaging and gadolinium-enhanced imaging as well, and these results were also included. In order to compare with initial imaging, MRI follow-up was obtained from 42/72 patients for at least 3 months. These images were then categorized into four subgroups: complete resolution, minor residuals (few remaining T2 signal changes, but much improved), moderate residuals (only minor improvement of T2 signal changes), and marked residuals (e.g., atrophy) (14).

### Statistical Analysis

The demographic, clinical, and radiological features of MOG-ab-positive and AQP4-ab-positive patients were compared using the χ^2^ test or Fisher exact tests for nominal data, while the Mann-Whitney U and Kruskal-Wallis tests were used for continuous variables. Relapse risk and disability outcome estimates were determined by Kaplan-Meier survival analysis and reported as median and (95% CI [confidence interval]). A univariate Cox proportional hazard model was used to compare time to event in AQP4 and MOG groups, and baseline covariates with a value of *p* ≤ 0.10 were included in the multivariate model.

All statistical analyses were performed with SPSS version 25.0 (IBM Corporation) and GraphPad Prism version 9.0 (GraphPad Software). Statistical significance was defined as a two-tailed *p* < 0.05.

## Results

### Demographics and Clinical Presentation

A total of 160 patients with ADS were included in the study. Although the number of patients who tested for different antibodies varied across the whole cohort, 64 of 135 (47.7%) patients were positive for MOG-ab, while AQP4-ab were found in 8 of 160 (5%) patients. When we evaluated 135 patients who were tested for both MOG-ab and AQP4-ab, the median age at onset was seven (interquartile range [IQR], 5–10) years, and the median follow-up period was 19 (IQR 13–27.5) months. Sixty-three patients (46.7%) were negative for both antibodies, and no patients had antibodies against both MOG and AQP4 antigens. Baseline features according to antibody status is shown in Supplementary Table 1.

Among the MOG-ab-positive patients, the male to female ratio was 1:1.2, and the median age at onset was seven (IQR 5–10) years. The most frequent phenotype at onset was ADEM (53.1%), followed by ON, which accounted for 21.9% of patients. The top three onset symptoms were encephalopathy (56.3%), motor dysfunction (53.1%), and vision impairment (45.3%). The clinical features of 13 (20.3%) patients with encephalitis other than ADEM included fever (11[84.6%]), seizures (5[38.5%]), brainstem and/or cerebellar involvement without symptoms of encephalopathy (5[38.4%]), and motor dysfunction (5[38.4%]). Pleocytosis and protein elevation in CSF were observed in 26/62 (41.9%) and 27/62 (43.5%) patients, respectively.

After a median follow-up of 16 (IQR 13–21) months, 19 of 63 (30.2%) patients had relapses. The frequency of relapse did not change according to the initial presentation (*p* = 0.612) or antibody variety (*p* = 0.130). Additionally, there were no differences in clinical recovery or MRI residuals among patients with different initial phenotypes (*p* = 0.280 and *p* = 0.520, respectively). At the last follow-up, 11(17.2%) patients fulfilled the criteria for NMOSD, 31(48.4%) patients fulfilled the criteria for ADEM, and 16 (25%) fulfilled the criteria for CIS. Only one patient who was positive for MOG-ab but negative for OCB was diagnosed with MS. Five out of the 13 patients who presented with encephalitis other than ADEM were finally diagnosed with uncategorized syndromes, and all of them experienced clinical relapse.

Table 1 summarizes the detailed demographic and clinical features based on final diagnosis in 135 children with both AQP4-ab and MOG-ab tested. When compared with the AQP4-ab-positive group, the MOG-ab-positive group was more likely to present with ADEM, but the difference was not statistically significant. AQP4-ab-positive patients were more likely to present with myelitis (*p* = 0.001), bladder or bowel dysfunction (*p* = 0.018), dyskinesias (*p* = 0.018), and paresthesia (*p* = 0.001) at onset. Although there were no differences in other serum autoantibodies between the two groups, AQP4-ab-positive patients were more likely to contain other autoantibodies. Clinical recovery was significantly better in MOG-ab-positive patients than in AQP4-ab-positive patients (*p* = 0.032) (Table 2).

**Table 1:** Demographic and clinical features of MOG-ab-positive, AQP4-ab-positive and both antibodies negative children according to their final diagnosis.

	**MS**	**NMOSD**	**ADEM**	**CIS**	**Uncategorized syndromes**
Patients	2	26	51	42	14
Age at presentation median (IQR), y	7 (4.5,9.5)	9 (6, 11)	6 (4,9.8)	7.5 (6, 10)	6 (4, 9)
Sex, M: F	0:2	1:1.9	1:1.1	2.2:1	1:1.3
Phenotype at onset, *n* (%)					
ADEM	1 (50)	7 (26.9)	48 (94.1)	0 (0)	1 (0)
Encephalitis (other than ADEM)	1 (50)	1 (3.8)	0 (0)	11 (26.2)	12 (85.7)
ON	0 (0)	8 (30.8)	3 (5.9)	19 (45.2)	0 (0)
Myelitis	0 (0)	7 (26.9)	0 (0)	12 (28.6)	1 (0)
ON with Myelitis	0 (0)	3 (11.5)	0 (0)	0 (0)	0 (0)
Abnormal brain MRI at onset, *n* (%)	1/1 (100)	19/23 (82.6)	48/50 (96)	25/37 (67.6)	14/14 (100)
AQP4-Ab, *n*	0	8	0	0	0
MOG-Ab, *n*	1	11	31	16	5
FU time, median (IQR), m	30 (20.5,39.5)	19 (16,25.8)	19 (13,28.2)	15 (11,20.8)	19 (16,28)

*Ab, antibody; ADEM, acute disseminated encephalomyelitis; AQP4, aquaporin−4; CIS, clinically isolated syndrome; EDSS, Expanded Disability Status Scale; FU, follow-up; IQR, interquartile range; MOG, myelin oligodendrocyte glycoprotein; MS, multiple sclerosis; NMOSD, neuromyelitis optica spectrum disorders; ON, optic neuritis*.

**Table 2:** Comparison of clinical characteristics in AQP4-ab-positive and MOG-ab-positive children.

	**AQP4-ab (+) (*n* = 8)**	**MOG-ab (+) (*n* = 64)**	***P* value**
Phenotype at onset, *n* (%)			
ADEM	1 (12.5)	34 (53.1)	0.073
Encephalitis (other than ADEM)	0 (0)	13 (20.3)	0.357
ON	3 (37.5)	14 (21.9)	0.589
Myelitis	4 (50)	2 (3.1)	0.001
ON with Myelitis	0 (0)	1 (1.6)	1.000
First attack symptoms, *n* (%)			
Seizure	1 (12.5)	13 (20.3)	0.958
Encephalopathy	2 (25)	36 (56.3)	0.196
Intractable nausea/ vomiting/ hiccups (APS)	4 (50)	24 (37.5)	0.765
Brainstem syndrome (excluding APS)	1 (12.5)	16 (25)	0.731
Cerebellar symptoms	2 (25)	16 (25)	1.0
Pyramidal signs	7 (87.5)	28 (43.8)	0.05
Bladder/bowel	6 (75)	17 (26.6)	0.018
Motor	8 (100)	34 (53.1)	0.031
Sensory	6 (75)	10 (15)	0.001
Vision	6 (75)	29 (45.3)	0.227
CSF findings^*a*^, *n* (%)			
CSF pleocytosis (>5 cells/mm3)	2/5 (40)	26/62 (41.9)	1.0
CSF protein elevation (>40 mg/dL)	2/5 (40)	27/62 (43.5)	1.0
Relapse, *n* (%)			
ON only	1/5 (20)	1/19 (5.3)	0.430
TM only	3/5 (60)	1/19 (5.3)	0.018
ON or TM with other	1/5 (20)	8/19 (42.1)	0.520
Other	0/5 (0)	9/19 (47.4)	0.105
Clinical recovery, *n* (%)			0.032
Complete	3/7 (42.9)	48/63 (76.2)	
Minor residuals	0/7 (0)	6/63 (9.5)	
Severe residuals	4/7 (57.1)	9/51 (14.3)	
EDSS score at onset, median (IQR)	4 (3.25, 5)	3 (1.38, 4)	0.071
EDSS score at 1 y, median (IQR)	3.5 (3.125, 3.875)	0 (0, 0)	0.002
EDSS score at 2 y, median (IQR)	3 (0,4)	0 (0, 0.5)	0.103
EDSS score at FU, median (IQR)	3 (0,3)	0 (0, 0)	0.062
Acute-phase therapy (IVMP/PLEX/IVIG)	7/8 (87.5)	62/64 (96.9)	0.301
Maintenance therapy at FU, *n* (%)	7/7 (100)	25/63 (39.7)	0.008

*APS, area postrema syndrome; Ab, antibody; ADEM, acute disseminated encephalomyelitis; AQP4, aquaporin−4; CSF, cerebrospinal fluid; EDSS, Expanded Disability Status Scale; FU, follow-up; IQR, interquartile range; IVIG, intravenous immunoglobulins; IVMP, intravenous methylprednisolone; MOG, myelin oligodendrocyte glycoprotein; ON, optic neuritis; PLEX, plasma exchange; TM, transverse myelitis; y, year(s)*.

a *Only lumbar puncture performed within one month from onset of symptoms are shown in bold*.

Of the 64 MOG-ab-positive patients, 40 patients had MOG-ab in both serum and CSF, 24 in serum alone. One patient had MOG-ab in CSF alone and was finally diagnosed with CIS. Of the 8 AQP4-ab-positive patients, 7 patients had AQP4-ab in both serum and CSF, 1 in serum alone. Only 5 of 64 (7.8%) MOG-ab-positive and 1 of 8 (12.5%) AQP4-ab-positive patients had OCB in the CSF, respectively. Two antibody-negative patients had OCB in the CSF, one of which was diagnosed with MS and another with ADEM.

In addition, 63 patients were negative for both antibodies from disease onset and were not found to be seroconverted to positive in the subsequent disease course, either relapsing or remission. Among them, 58.7% were male, and the median age at onset was 7 (IQR, 4.5–10) years. In this cohort, 11 of 57 (19.3%) patients relapsed after a median follow-up of 19 months (IQR 13–28 months). Only 14 of 56 (25%) patients still used maintenance therapy at the last follow-up. The parents of a patient diagnosed with uncategorized syndromes decided to give up on treatment due to persistent disturbance of consciousness and the patient died shortly after discharge (the exact cause of death was unknown).

### Radiological Features

Cerebral MRIs were available in 59 MOG-ab-positive patients, and 54 (91.5%) were abnormal at onset. Thirty-four of 59 (57.6%) patients presented with a pattern of poorly demarcated lesions, but only 11 of them had confluent lesions in both gray and white matter. Extensive, confluent, and essentially symmetric white matter (leukodystrophy-like) lesions were observed in five patients. Five patients also had sharply demarcated, hemispheric white matter lesions (>3 cm), but none were diagnosed with MS. Nonspecific white matter lesions were seen in patients with the initial phenotype of ON (53.8%) or TM (100%). Moreover, three patients presented with cortical gray matter lesions, similar to the pattern of cortical encephalitis changes. Among these three patients, one had unilateral changes and fully recovered at the last follow-up, and one had gadolinium enhancement combined with a tumefactive lesion of the cerebellum. One patient showed a linear leptomeningeal enhancement pattern. Interestingly, we found that two patients with intracallosal lesions were not compatible with periventricular lesions simultaneously. They were not diagnosed with MS but were finally diagnosed with ADEM, and their onset age was only 3 years.

The brain MRI findings in eight out of the 13 patients with encephalitis other than ADEM were not compatible with ADEM: two had isolated cortical or cortical-subcortical single lesions, one had extensive bilateral cortical involvement with additional basal ganglia changes, two had isolated unilateral thalamic or basal ganglia involvement, one had isolated brainstem involvement, one had isolated cerebellum and cerebellar peduncle involvement, and one had meningeal enhancement similar to meningitis. One patient had a leukodystrophy-like lesion and four patients had poorly demarcated deep white lesions, but none demonstrated clinical encephalopathy at onset.

Spinal MRI was performed in 29 MOG-ab-positive patients at onset. Eight of these patients (27.6%) had longitudinally extensive transverse myelitis (LETM), defined as myelitis extending three or more spinal segments, five had a short transverse myelitis, and approximately half (52.7%) had normal images. However, only five of the eight patients with LETM and two of the five patients with short transverse lesions were clinically consistent with transverse myelitis. The lesions showed diffuse central T2 hyperintensity with predominant swelling that was not confined to the gray matter on axial sequences. Orbital MRI was performed in 17 patients, of whom 11 (64.7%) were abnormal. Bilateral lesions were found in nine of these 9 (81.8%) patients, and pre-chiasmal segment involvement was the most common, as noted in eight images (72.7%). Only one patient had clinically silent optic nerve lesions, and all the remaining patients had optic nerve lesions with clinical optic neuritis.

**Figure 2:**
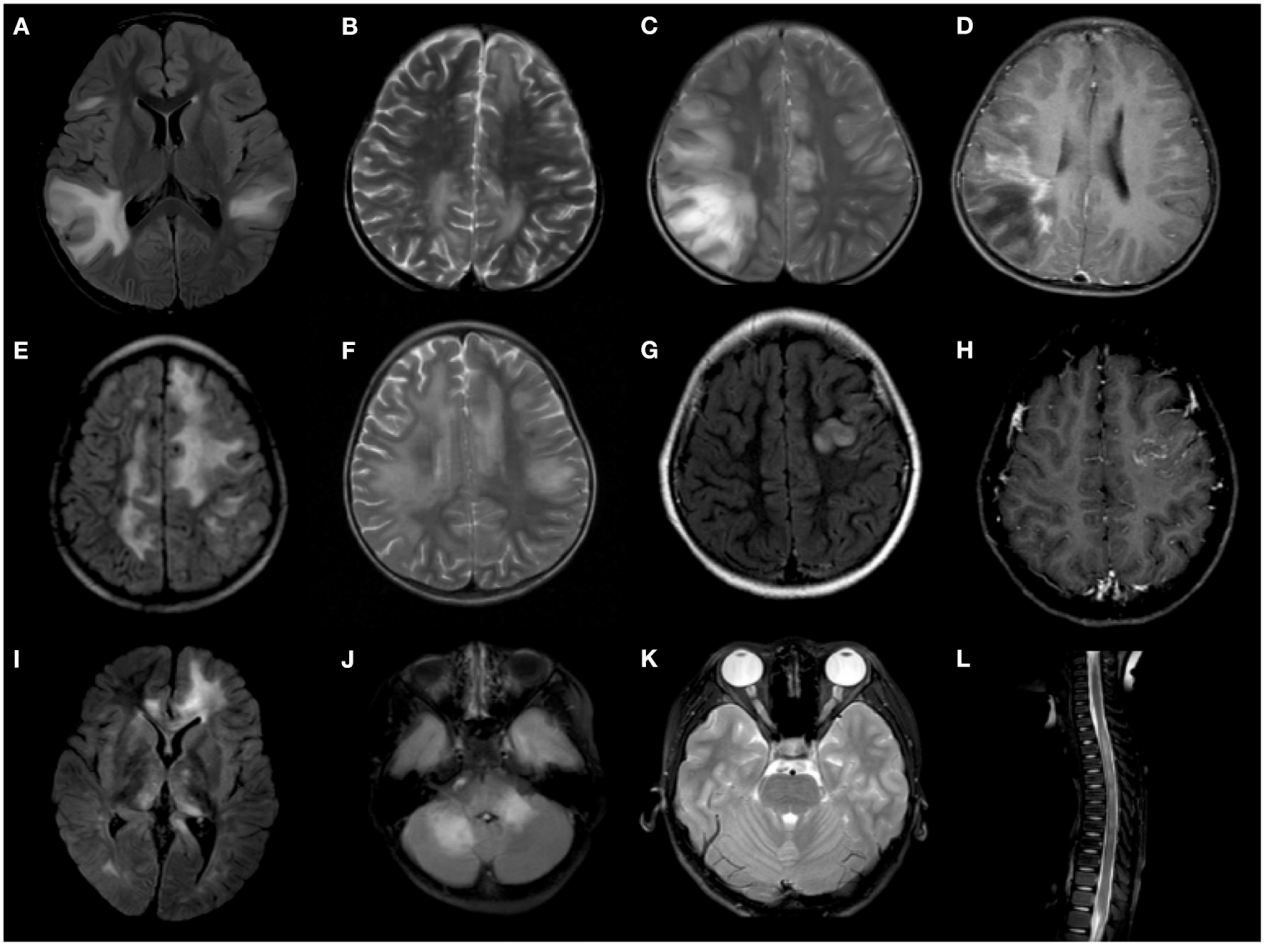
Representative radiological features of MOG-ab-positive patients. **(A–D)** Confluent, poorly demarcated lesions in gray and white matter. **(E,F)** Extensive, confluent, essentially symmetric white matter lesions (leukodystrophy-like). **(G,H)** Unilateral cortical lesion. **(I)** Deep gray matter involvement. **(J)** Cerebellum and cerebellar peduncle lesions. **(K)** Bilateral optic nerve involvement. **(L)** longitudinally extensive transverse myelitis (LETM).

MOG-ab-positive patients tended to have bilateral lesions and deep gray matter involvement more often than AQP4-ab-positive patients (*p* = 0.032 and *p* = 0.038, respectively) on brain MRI. However, spinal involvement was more common in AQP4-ab-positive patients than in MOG-ab-positive patients (*p* = 0.027), and cervical and thoracic segment involvement was more distinct on spinal MRI (*p* = 0.018 and *p* = 0.008, respectively). However, there were no differences in the location and enhancement between the two cohorts on orbital MRI (Table 3). Figure 2 shows representative radiological images of MOG-ab–positive patients.

**Table 3:** Comparison of radiologic features in AQP4-ab-positive and MOG-ab-positive children.

	**AQP4-ab (+) (*n* = 8)**	**MOG-ab (+) (*n* = 64)**	***P* value**
Cerebral lesions at onset, n (%)	7	59	
Abnormal	6 (85.7)	54 (91.5)	0.504
Bilateral	1 (14.3)	38 (64.4)	0.032
Cortical GM	0 (0)	11 (18.6)	0.556
Juxtacortical WM	2 (28.6)	28 (47.5)	0.584
Deep WM	2 (28.6)	20 (33.9)	1.000
Periventricular WM	2 (28.6)	13 (22)	1.000
Deep GM	1 (14.3)	29 (49.2)	0.038
Thalamus	0 (0)	22 (37.3)	0.120
Basal ganglia	1 (14.3)	25 (42.3)	0.304
Brainstem	1 (14.3)	23 (39)	0.532
Pons	0 (0)	18 (30.5)	0.385
Midbrain	0 (0)	19 (32.2)	0.206
Medulla	1 (14.3)	2 (3.4)	0.290
Area postrema	1 (14.3)	0 (0)	0.106
Intracallosal	1 (14.3)	2 (3.4)	0.290
Cerebellum	0 (0)	17 (28.8)	0.234
Cerebellar peduncle	0 (0)	12 (20.3)	0.404
Adjacent to 4th ventricle	0 (0)	9 (15.3)	0.581
Enhancement	0 (0)	8 (13.6)	0.584
Spinal MRI at onset, n (%)	7	29	
Abnormal	7 (100)	13 (44.8)	0.027
LETM	5 (71.4)	8 (27.6)	0.073
Length of LETM, median (range) TM	16 (15,16)	8 (3,13)	0.019
Cervical involvement	5 (71.4)	6 (20.7)	0.018
Thoracic involvement	6 (85.7)	8 (27.6)	0.008
Lumbar involvement	0 (0)	3 (10.3)	1.000
Conus involvement	0 (0)	2 (6.9)	1.000
Orbital MRI, n (%)	4	17	
Abnormal	3 (75)	11 (64.7)	1.000
Bilateral	3 (75)	9 (52.9)	0.603
Pre-chiasmal	1 (25)	8 (47)	0.603
Canalicular segment	1 (25)	6 (35.3)	1.000
Orbital segment	0 (0)	3 (17.6)	0.539
Cranial segment	2 (50)	5 (29.4)	0.574
Optic chiasm	1 (25)	3 (17.6)	1.000
Optic tracts	0 (0)	2 (11.8)	1.000
FU time, median (IQR), month	12 (7,18)	16 (16,17)	0.146
MRI recovery	5	42	0.006
Complete	0 (0)	15 (35.7)	
Mild residuals	0 (0)	11 (26.2)	
Moderate residuals	1 (20)	8 (19)	
Severe residuals	4 (80)	8 (19)	

*Ab, antibody; AQP4, aquaporin-4; FU, follow-up; GM, gray matter; LETM, longitudinal extensive transverse myelitis; IQR, interquartile range; MOG, myelin oligodendrocyte glycoprotein; NA, not available; ON, optic neuritis; TM, transverse myelitis; WM, white matter*.

Serial MRIs were available in 42 MOG-ab-positive patients during a median follow-up period of 12 months (IQR 7–18), which revealed complete resolution in 15 of 42 (35.7%) patients, mild/moderate residuals in 17 of 42 (40.5%) patients, and severe residuals in 8 of 42 (19%) patients. Compared with 5 AQP4-ab-positive patients with serial MRIs during follow-up, MRI recovery was better in MOG-ab-positive patients (*p* = 0.006), and only four patients experienced severe residuals. Additionally, patients who did not relapse during follow-up had a significant resolution of lesions (*p* = 0.004) compared with patients who relapsed. Three of the eight patients with severe residuals on MRI had a clinically complete recovery, whereas three of the 15 patients with a complete resolution on MRI had severe residuals in clinical symptoms at the last follow-up.

### Therapeutic Response and Clinical Outcomes

#### Maintenance Corticosteroids Usage and Relapse Risk

**Figure 3:**
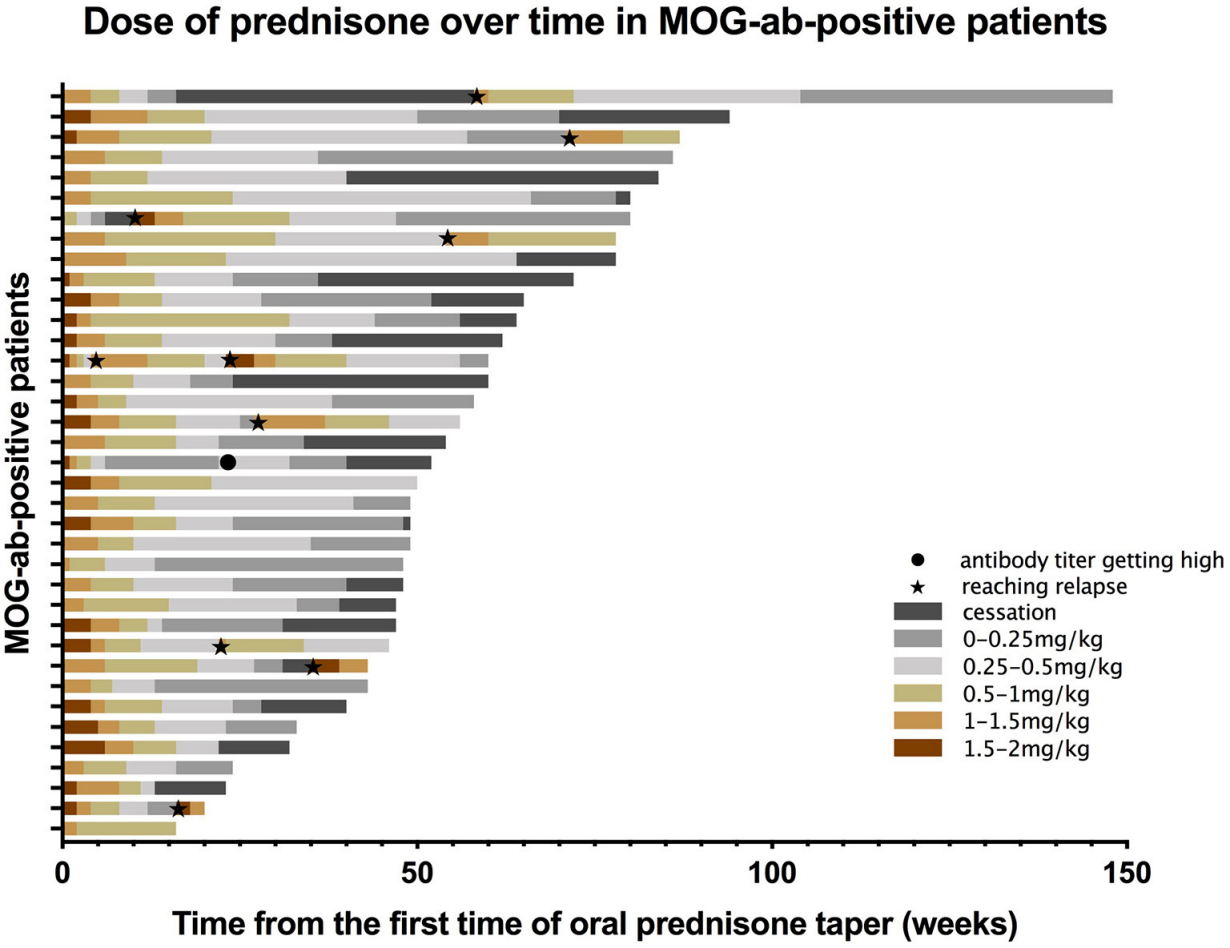
Prednisone taper dose over time in MOG-ab-positive patients. Each bar represents an individual participant. Different colors represent different doses of prednisone. For example, reddish-brown bars indicate the taper for 1.5–2 mg/kg per day. The black dot indicates a sudden high titer of MOG-ab in a patient, and black pentagrams indicate patients reaching relapse.

When we assessed treatment data on disease presentation, we found 61 MOG-ab-positive patients were treated with corticosteroids, and 53 were treated with intravenous methylprednisolone (IVMP) in the acute phase following oral tapering with prednisone. Five patients were treated with oral corticosteroids alone at onset. Comprehensive data on oral prednisone usage were available for 48 episodes. The median duration of oral tapering was 38 weeks, other than usage without cessation. Except for 10 relapsing episodes and one episode with a suddenly elevated antibody titer, the use of prednisone was collected for all treated patients (Figure 3). The median length of oral tapering for 1–2 mg/kg daily was five (IQR 4–8) weeks, following 0.5–1 mg/kg daily over seven (IQR 5–10) weeks, 0.25–0.5 mg/kg daily over 12 (IQR 7–26.5) weeks, and from 0.25 mg/kg daily to cessation over 12 (IQR 8–20) weeks.

**Figure 4:**
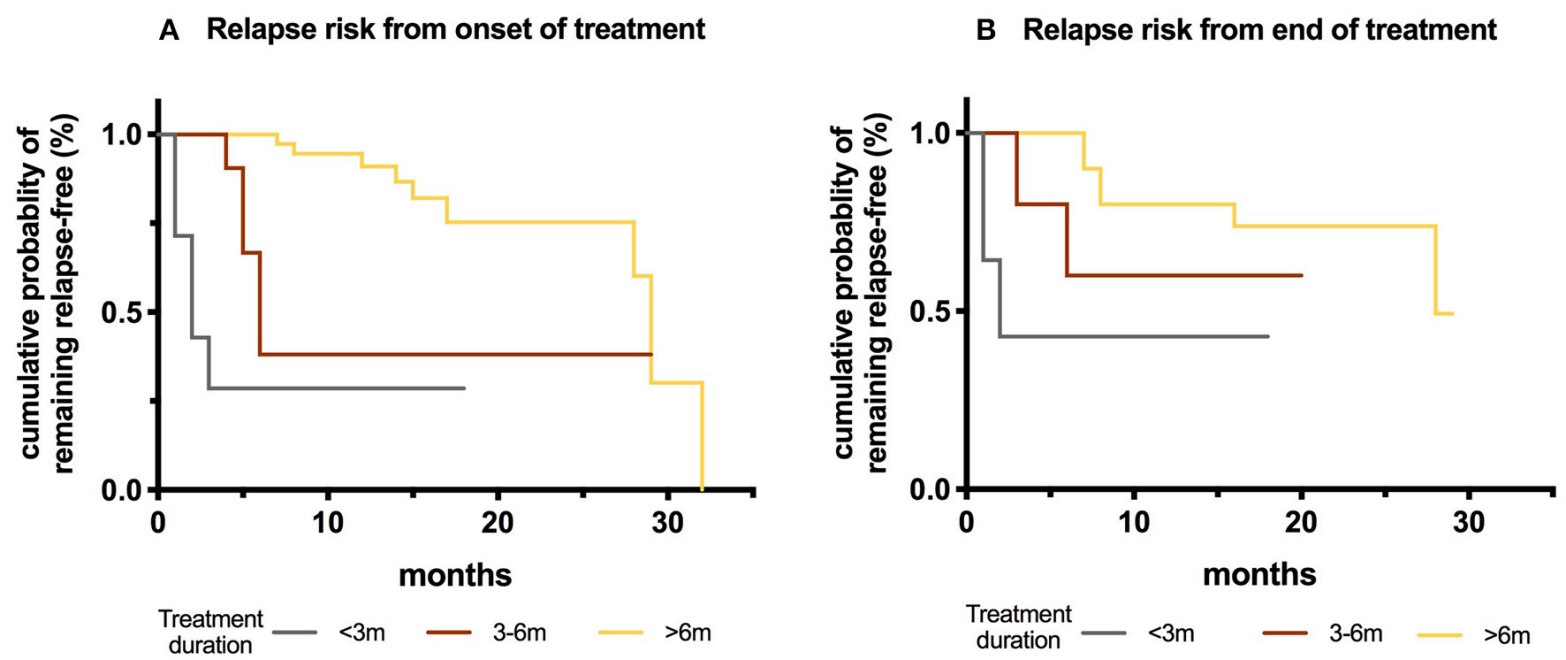
Kaplan-Meier curves showing risk of relapse depending on the duration of oral prednisone taper in MOG-ab-positive patients. **(A)** Treatment duration from onset of disease: <3 months, between 3 and 6 months and >6 months. **(B)** Observation time after the treatment cessation.

We then assessed subsequent relapses during oral prednisone weaning, including 31 episodes, most of which (19/31) occurred toward the cessation of taper, or shortly after cessation (10/18, some data were not available). The risk of relapse was higher in patients who were treated for less than 6 months in comparison to those treated for more than 6 months (*P* < 0.001), and in patients who stopped prednisone within three months in comparison to those who stopped treatment for more than six months (*p* = 0.013) (Figure 4).

Even though the dose of prednisone at the time of relapse was not different between the two cohorts (*p* = 0.712), all the AQP4-ab-positive patients relapsed within five months of prednisone cessation, which was different from MOG-ab-positive patients (*p* = 0.028) (Supplementary Figure 1).

#### Other Immunosuppressive Treatments

Eight MOG-ab-positive patients only received intravenous immunoglobulin during the acute phase of disease presentation, and one of them relapsed during follow-up (one was withdrawn). Forty-two patients received both intravenous immunoglobulin (IVIG) and IVMP, but 10 relapsed. No patients in our study were subsequently prescribed IVIG for monthly infusion. Table 4 summarizes those who had other immunosuppressive agents for more than 6 months. The time from the initial attack to the administration of therapy was 17(IQR 1–58) months. In addition, one patient received AZA shortly after diagnosis and did not relapse; the other seven patients were commenced on immunosuppressive agents after clinical relapses. Six of eight patients did not relapse after treatment, and one of them switched treatment from RTX to MMF due to adverse symptoms, but ultimately did not relapse. Taking all agents into account in the MOG-ab-positive group, ARRs during treatment were significantly improved from before treatment administration (*p* = 0.007).

**Table 4:** Efficacy of other immunosuppressive agents in MOG-ab-positive children.

**Treatment modality**	**Number of patients on treatment for ≥6 months**	**Therapy duration (months)**	**Disease duration before administration of the therapy (months)**	**ARR before treatment**	**ARR during treatment**	**Change of ARR before and on treatment, %**	**Wilcoxon *p* value**
RTX	2	15	4	4	0	100	0.333
		17	29	1.24	0.71	42.7	
MMF	5	19	58	0.62	0.63	−1.6	0.016
		10	7	3.43	0	100	
		6	40	1.2	0	100	
		6	7	2	0	100	
		28	27	0.88	0	100	
AZA	1	6	1	0	0	/	/
Total, median (range)	8	12.5 (6–28)	17 (1–58)	0 (0-4)	0 (0-0.71)	100 (-1.6-100)	0.007

*ARR, annualized relapse rate; AZA, azathioprine; MMF, mycophenolate mofetil; RTX, rituximab*.

*The Wilcoxon signed-rank test was used to identify differences between the ARR before treatment and on-treatment*.

#### Risk Factors of Outcomes

**Figure 5:**
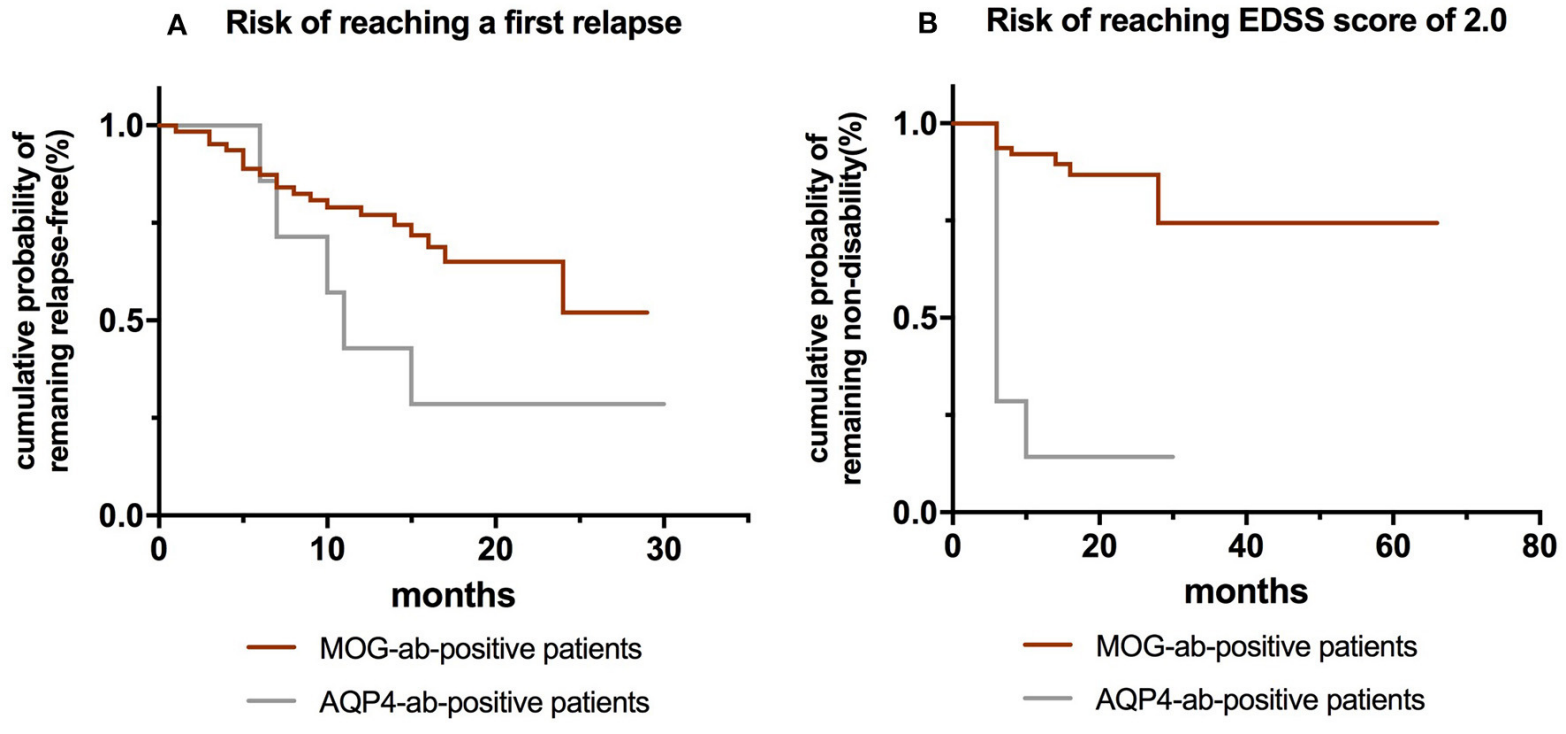
**(A)** Kaplan-Meier curves showing risk of reaching a first relapse between MOG-ab-positive patients and AQP4-ab-positive patients. **(B)** Kaplan-Meier curves showing risk of reaching EDSS 2.0 between MOG-ab-positive patients and AQP4-ab-positive patients.

Table 5 shows the influences of baseline epidemiological, clinical, and CSF characteristics on time-to-event in both MOG-ab-positive and AQP4-ab-positive groups. MOG-ab-positive patients trended toward a lower risk of first relapse and a better outcome compared to AQP4-ab-positive patients (Figure 5). To correct the deviation of outcomes, we included age at onset in both multivariate models. We found that maintenance therapy (hazard ratio [HR] 0.376,95% CI 0.145–0.971, *p* = 0.043) reduced the risk of clinical relapse, and presenting with ON at onset (HR 2.996,95% CI 1.176–7.628, *p* = 0.021) was related to a higher risk of relapse (Supplementary Figure 2). A higher EDSS score at onset (HR 1.722, 95% CI 1.169–2.537, *p* = 0.006) was also related to a worse outcome.

**Table 5:** Effects of baseline epidemiologic, clinical and CSF characteristics on time to reach a first relapse and time to reach EDSS score of 2.0.

**Basal variables**	**Time to reach a first relapse**		**Time to reach EDSS score of 2.0**	
	**Univariate**	**Multivariate**	**Univariate**	**Multivariate**
MOG-ab serostatus	0.422 (0.157–1.234)	0.615 (0.199–1.903)	0.110 (0.037–0.329)	0.348 (0.093–1.310)
	0.087	0.399	<0.001	0.119
Age	0.926 (0.819–1.047)	0.875 (0.764–1.001)	1.133 (0.956–1.343)	0.930 (0.753–1.147)
	0.222	0.051	0.148	0.497
Female	0.583 (0.241–1.406)		1.251 (0.419–3.736)	
	0.230		0.688	
Presenting ON at onset	2.650 (1.103–6.363)	2.996 (1.176–7.628)	1.789 (0.0.599–5.344)	
	0.029	0.021	0.298	
maintain therapy	0.378 (0.156–0.915)	0.376 (0.145–0.971)	0.171 (0.038–0.764)	0.276 (0.055–1.375)
	0.031	0.043	0.021	0.116
CSF pleocytosis	0.659 (0.271–1.605)		0.970 (0.317–2.967)	
	0.359		0.958	
EDSS score at onset	0.282 (0.714–1.103)		1.759 (1.302–2.374)	1.722 (1.169–2.537)
	0.282		<0.001	0.006

*CSF, cerebrospinal fluid; EDSS, Expanded Disability Status Scale; MOG-ab, myelin oligodendrocyte glycoprotein antibody; ON, optic neuritis*.

*In univariate and multivariate analyses, data are given as follows: hazard ratio (95% confidence interval), p value*.

### Serial Measurements of MOG-abs

Serial MOG-ab serostatus was available in 42 patients over the course of the disease. Of note, no patients were MOG-ab seronegative during relapse. After a median follow-up period of 13 (IQR 10–15) months, 26 patients remained persistently seropositive, and nine (34.6%) had clinical relapses. Six patients were monophasic despite persistently rising MOG-ab titers. Thirteen (31%) patients who experienced a median follow-up period of 10 months (IQR, 4–14 months) converted to seronegative. All of them had no further clinical attacks after a median follow-up of three (IQR 2–6) months, which is significantly different from patients with persistent seropositivity (*p* = 0.018). Three patients were still on maintenance treatment due to residual MRI lesions, while others stopped treatment soon after the detection of seroconversion. Furthermore, there were three (7.1%) patients with a reoccurrence of MOG-ab after initial seroconversion from positive to negative serostatus, one of which experienced clinical relapse.

We found that the frequency of seroconversion was not related to the initial presenting phenotype (*p* = 0.563), but the final diagnosis was significantly different (*p* = 0.028). Of note, the four patients who were finally diagnosed with uncategorized syndromes developed seroconversion. Meanwhile, there was no difference in the interval of seroconversion to negative among children with different diagnoses (Table 6).

**Table 6:** Comparison of demographic and clinical features of children with MOG-ab persistently seropositive and seroconverted to negative.

	**Persistent seropositivity**	**Seroconverted to negative**	***P* value**
Numbers of patients	26	13	/
Sex, M: F	7:6	3:10	0.093
Age at presentation median (IQR), y	6.5 (5,9)	6 (5,9)	0.803
Presenting phenotype at onset, *n* (%)			0.563
ADEM	14 (53.8)	6 (46.2)	0.741
Encephalitis (other than ADEM)	4 (15.4)	4 (30.8)	0.402
ON	6 (23)	3 (23.1)	1.000
Myelitis	2 (7.7)	0 (0)	0.544
ON with Myelitis	0 (0)	0 (0)	NA
Final diagnosis, *n* (%)			0.028
NMOSD	5 (19.2)	1 (7.7)	0.643
ADEM	14 (53.8)	6 (46.2)	0.741
CIS	7 (26.9)	2 (15.4)	0.689
Uncategorized syndromes	0 (0)	4 (30.8)	0.009
Relapse, *n* (%)	9 (34.6)	0 (0)	0.018
EDSS score at onset, median (IQR)	2.5 (1.125,4)	2 (0,4)	0.848

*Ab, antibody; ADEM, acute disseminated encephalomyelitis; CIS, clinically isolated syndrome; EDSS, Expanded Disability Status Scale; IQR, interquartile range; NA, not available; NMOSD, neuromyelitis optica spectrum disorders; ON, optic neuritis*.

## Discussion

In this study of 160 children with ADS, we evaluated the proportion of patients with various antibody serostatuses. Our cohort proved that MOG-abs remain in the majority of children with ADS (16), but yielded results inconsistent with previous reports (16, 17) in which MOG-abs were detected in more than 40% of children with ADS. The lack of MOG-ab detection in the early years reduced the total number of tested children which may result in an increased positive rate of MOG-ab. The 30% recurrence rate in MOG-ab-positive patients reflects the results of other studies (16, 18). Jarius et al. (19) reported a multiphasic course in 80% of MOG-ab-positive patients in a subset of the NMOSD adult cohort. Since patients in our cohort who experienced clinical relapses had different follow-up times when compared with patients who had monophasic disease duration (*p* = 0.001), it is plausible that the recurrence rate may be overestimated with the extension of follow-up time, or patients without serostatus at onset were inclined to test antibodies only if they reached relapse, thereby increasing the recurrence rate. Given that some seronegative patients were found seropositive for antibodies after recurrence, a longer observation was needed to prove the prognosis of the group.

MOG-ab-positive patients, either at onset or throughout the course, are accompanied by ON (20–23), predominantly in adolescents (7, 18). The relative levels of regional expression of MOG antigen vary among different age groups to some extent, which indicates an age-dependent phenotype in MOG-ab-positive patients (20–23). In our study, ADEM had a striking preponderance in clinical presentation compared to ON which may result from the younger onset age of patients in our cohort. In a study comparing pediatric ADEM patients with and without MOG-abs, 19 MOG-ab-positive patients demonstrated a distinct MRI pattern characterized by blurred bilateral widespread lesions without clear borders and more obvious spinal cord involvement, and they were less likely to present emotional and behavioral symptoms (14). Wegener-Panzer et al. (24) reported 10 children with autoimmune encephalitis associated with MOG-abs who showed a radiologic pattern of involvement of the cortical region with absent diffusion restriction and contrast enhancement. Similarly, patients with uncategorized syndromes in our cohort also presented with atypical MRI features, such as unilateral cortical lesions and meningeal enhancement lesions. Unlike the previous study of patients with encephalitis other than ADEM (16) who had worse outcomes, patients with these phenotypes in our cohort all became MOG-ab seronegative and reached full recovery at last follow-up despite the relapsing course. These patients may have another autoantibody as a causative agent that accounts for additional demyelinating features (9, 10). A study of seronegative patients who had MOG-abs in CSF identified no common characteristics in those patients except for the absence of isolated ON (25). In our cohort, the seronegative patient with MOG-abs in CSF was presented with bilateral ON, which filled the data gap. Therefore, regardless of whether the MOG-ab harbored by such patients is an epiphenomenon, it is undeniable that screening both serum and CSF specimens for MOG-abs should be considered in pediatric patients with other atypical autoimmune encephalitis as well as seronegative cases with high clinical suspicion.

In our study, symptoms of myelitis were more predominant in patients with AQP4-abs than in those with MOG-abs either at onset or relapse, but LETM did not help distinguish between the two groups, similar to other reports (26, 27). None of the five patients initially presenting with a short segments spinal lesion were diagnosed with MS as prior reported (7). However, spinal cord involvement may have been underestimated because patients with asymptomatic spinal lesions may not perform spinal imaging immediately. Although lumbar and conus involvement were unique to MOG-ab-positive patients (26, 27), a Chinese report (28) was consistent with our study, outlining that conus involvement was not common when compared with AQP4-ab-positive patients, which may be related to race. Our study did not find a novel T2 signal confined to gray matter (sagittal T2 hyperintensity line and axial H pattern sign) favoring MOG-abs as previously reported (27), which may be due to the young age of our cohort. In cerebral images, except that deep gray matter nuclei were more common in MOG-ab-positive patients, overlapping features could be seen between the two groups, as recently suggested (21, 29). However, a high frequency of AQP4 distribution locations such as periependymal, periaqueductal, and hypothalamic were shown more frequently in AQP4-ab-positive patients in one study (26), along with the area postrema and the dorsal brainstem in another study (6).

At present, the treatment protocols for patients with MOG-abs are mostly based on limited retrospective research and treatment experience from other autoimmune diseases, such as AQP4-ab-positive NMOSD. In a UK study, immunosuppressive treatment for more than 3 months following the onset of an attack was associated with a lower risk of relapse in MOG-ab-positive patients (20). In our study, the borderline time for the use of prednisone to reduce recurrence risk was 6 months, and patients were vulnerable to relapse at a minimal dosage, which was concordant with a prior study (23). However, there were still a small number of patients who had clinical symptom resurgence without immunotherapy for a long time after initiating treatment. Treatment with RTX appeared to reduce ARRs and stabilize EDSS in pediatric cohorts with MOG-ab-positive patients, most of whom were having a relapsing course (30, 31). Other studies also showed the efficacy of MMF in reducing relapse risk (23, 31, 32), whereas Ramanathan et al. (23) reported that MMF reduced relapse risk exclusively when used with steroids. Even if our data were sparse on other immunosuppressive treatments and some patients were using steroids concomitantly, RTX and MMF were still effective in reducing relapses.

Irrespective of the relapse course, the presence of MOG-abs is still associated with a moderate relapse risk and is a predictor of a favorable outcome (14, 21). A previous study in AQP4-ab-positive NMOSD (33) found that Japanese patients and immunosuppressant drugs were associated with a lower risk of recurrence attacks, while female sex and MS disease-modifying agents increased the likelihood of relapse. In MOG-ab-positive patients, the occurrence of ON appeared to have a protective effect on sustained disability, while TM was a predictor of sustained residual deficits (20, 23).

When combining the different factors in these two individual groups, our data suggested that maintenance therapy indicated a lower risk of clinical relapse, which reinforces other reports (21). In patients with recurrent ON, MOG-abs were associated with a higher relapse rate, and the interval time between initial and second attacks was shorter (34), which supported our finding that presenting with ON was an independent risk factor for relapse. We also found that the EDSS score at onset seemed to be a useful predictor of clinical outcomes. Although we did not differentiate between types of disability, and the EDSS score is weighted in favor of motor impairment over other impairments (such as visual and cognitive disability), most disabilities were driven by attack onset, as prior observations (20–22) have suggested that clinical phenotypes at onset may predict the eventual type of disability accordingly.

Although the relationship between AQP4-ab level and disease severity was unclear, AQP4-ab-positive patients may be at risk of clinical recurrence, and prevention treatment should be considered even in the case of prolonged remission. Several studies on MOG-ab-positive patients found that patients with persistent MOG-ab seropositivity after treatment were more likely to relapse, and their initial antibody titers were also higher (18, 20, 35). Our data showed no clinical relapse in patients with seroconversion, which is consistent with other reports (20, 35). Patients who were diagnosed with uncategorized syndromes seemed to be more likely to develop seroconversion. Similar to the results identified previously (36, 37), some MOG-ab-positive patients had a clinical relapse with serum antibody fluctuations. Some children had relapse-free periods despite persistently rising titers (36).

After collecting steroid usage in MOG-ab-positive patients, this study proposes a summarized prednisone taper scheme as follows: patients will commence the oral taper after acute therapy, receiving prednisone at 1–2 mg/kg daily for about 1 month, 0.5–1 mg/kg daily for about 2 months, and 0.25–0.5 mg/kg daily for about 3–4 months, and from 0.25 mg/kg daily to cessation for about 3–4 months. The recommended duration of the entire treatment was more than 6 months. Serial MRI scans and the serological surveillance of MOG-ab are essential to decide further treatment in practical situations. Recently, E.U. pediatric MOG consortium consensus proposed a recommendation for steroid tapering with oral prednisone after acute treatment for <3 months to avoid side effects (38). A study reported by Waters et al. (37) also considered that commencing long-term immunomodulatory therapy for patients immediately following their first episodes was not appropriate because 72% of persistently seropositive patients in their study remained monophasic at the last follow-up. Nevertheless, patients presenting with ON seemed to be treated more aggressively because 8% developed visual disability after recovery (20) and relapsed earlier in our study. Constrained by our retrospective design and some patients utilizing multiple agents simultaneously, we may not have found the optimal condition and timing of maintenance options on this occasion.

Our study has several limitations. The most important limitation was that some patients were only tested for AQP4-ab but not for MOG-ab at initial presentation, which might impact the accuracy of antibody positivity. The small cohort size and relatively short follow-up time restricted our ability to obtain significant findings and apply them to larger groups. Third, owing to the retrospective nature of our study, we were unable to evaluate the precise relationship between serum titers and relapse time. Meanwhile, serum samples for detection according to a certain time interval would be difficult to implement clinically.

## Conclusion

MOG-abs are more common in children with ADS than AQP4-abs. MOG-antibody-associated disorders had wider disease phenotypes than the initial spectrum, with distinct clinical, radiological, and immunologic features. Although some MOG-ab-positive children experience relapsing courses and persistent seropositive status, they predict a better outcome than AQP4-ab-positive patients. It is recommended that steroid maintenance therapy is used for MOG-ab-positive children, but future multicenter and controlled studies are needed to mitigate other baseline differences and indicate bias to justify its long-term safety and efficacy.

## Data Availability Statement

The original contributions presented in the study are included in the article/Supplementary Material, further inquiries can be directed to the corresponding author/s.

## Ethics Statement

Written informed consent was obtained from the minor(s)' legal guardian/next of kin for the publication of any potentially identifiable images or data included in this article.

## Author Contributions

ZL and XL contributed to the conception and design of the study with suggestions from LJ, PW, MZ, and JM. ZL, HS, PY, and YJ collected and analyzed the data. XF performed the radiologic analysis. ZL drafted the initial manuscript and edited by LJ and XL. All authors approved the final version of the manuscript.

## Conflict of Interest

The authors declare that the research was conducted in the absence of any commercial or financial relationships that could be construed as a potential conflict of interest.

## Supplementary Material

Supplementary Figure 1 An illustration of the proportion of recurrence in MOG-ab-positive patients and AQP4-ab-positive patients at different doses of prednisone, or different time from cessation of the oral taper.

Supplementary Figure 2 Kaplan-Meier curves showing time to first relapse. **(A)** All MOG-ab-positive patients and AQP4-ab-positive patients. The 95% confidence interval is shown in gray. **(B)** Depending on the onset phenotypes. There is a trend toward patients presenting with ON at onset relapsing sooner than other patients.

Supplementary Table 1 Baseline clinical features according to antibodies status in 135 participants.

## Abbreviations

ADEM: acute disseminated encephalomyelitis
ADS: Acquired demyelinating syndromes
AQP4: aquaporin-4
ARR: annualized relapse rate
CIS: clinically isolated syndrome
EDSS: Expanded Disability Status Scale
LETM: longitudinally extensive transverse myelitis
MOG: myelin oligodendrocyte glycoprotein
MS: multiple sclerosis
NMOSD: neuromyelitis optica spectrum disorders
OCB: oligoclonal bands
ON: optic neuritis.
