# Supplementary figures and images for: Clinical and Prognostic Analysis of Autoantibody-Associated CNS Demyelinating Disorders in Children in Southwest China
Source: Front Neurol. 2021 Mar 26;12:642664. doi: 10.3389/fneur.2021.642664 (PMC8033000; doi:10.3389/fneur.2021.642664)

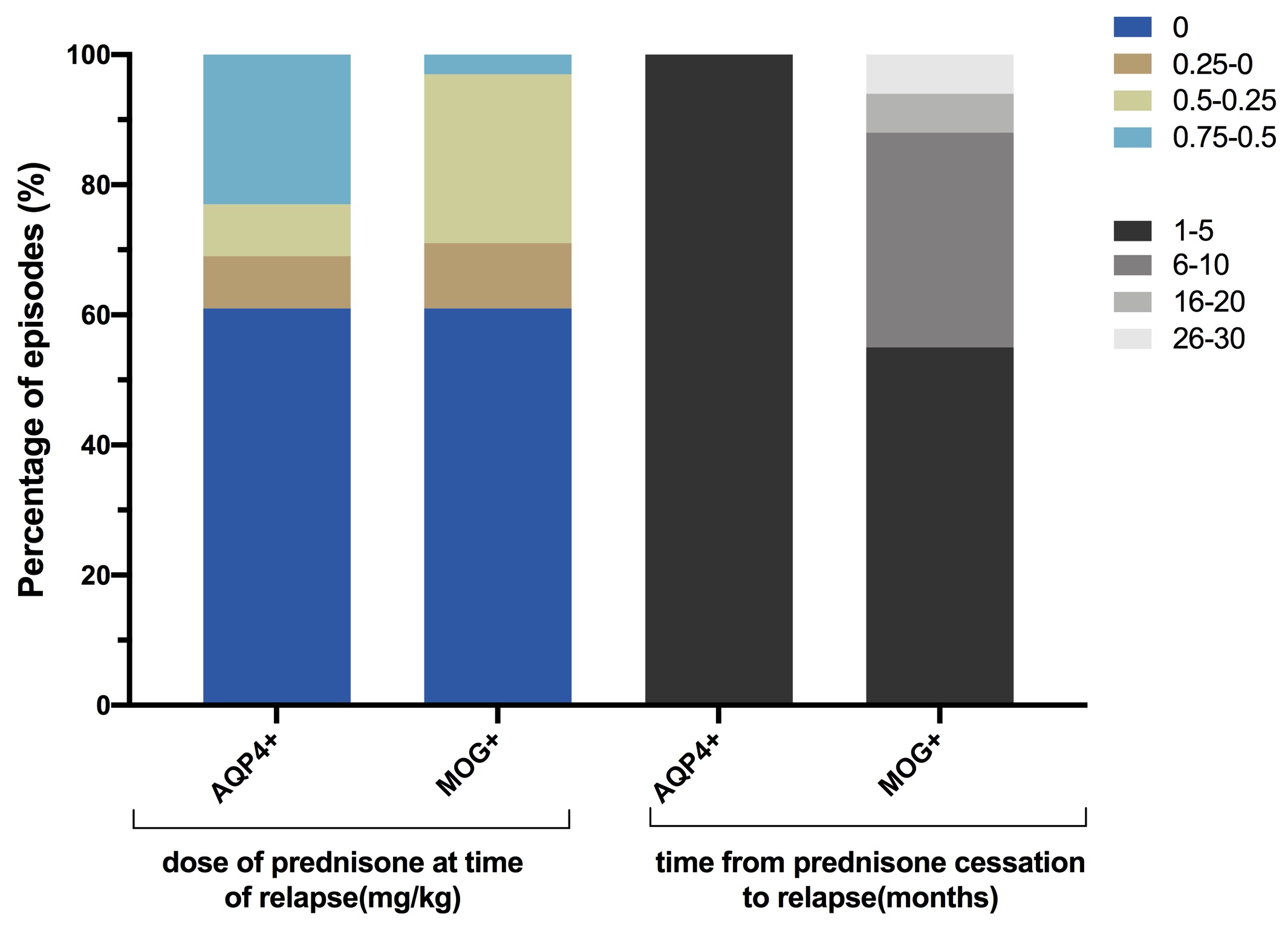

Supplement: Supplementary Figure 1 — An illustration of the proportion of recurrence in MOG-ab-positive patients and AQP4-ab-positive patients at different doses of prednisone, or different time from cessation of the oral taper. [file Image_1.JPEG]

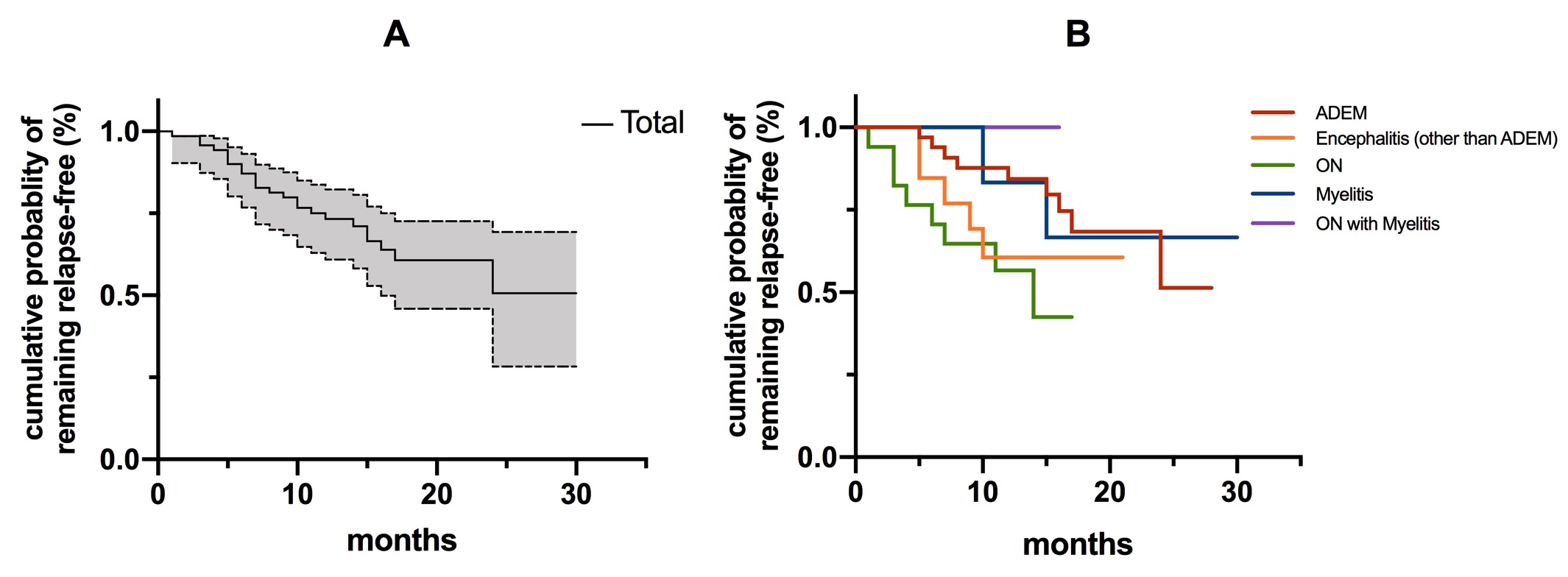

Supplement: Supplementary Figure 2 — Kaplan-Meier curves showing time to first relapse. (A) All MOG-ab-positive patients and AQP4-ab-positive patients. The 95% confidence interval is shown in gray. (B) Depending on the onset phenotypes. There is a trend toward patients presenting with ON at onset relapsing sooner than other patients. [file Image_2.JPEG]
