# Supplementary material for: Clinical and Prognostic Analysis of Autoantibody-Associated CNS Demyelinating Disorders in Children in Southwest China
Source: Front Neurol. 2021 Mar 26;12:642664. doi: 10.3389/fneur.2021.642664 (PMC8033000; doi:10.3389/fneur.2021.642664)
Supplement: Supplementary Table 1 — Baseline clinical features according to antibodies status in 135 participants. [file Table_1.docx]

Supplementary table 1. Baseline clinical features according to antibodies status in 135 participants

|  | AQP4-Ab (+) | MOG-Ab (+) | Both antibodies negative |
| --- | --- | --- | --- |
| Number of patients | 8 | 64 | 63 |
| Male: Female | 1:3 | 1:1.2 | 1.4:1 |
| Age at presentation, median (IQR), y | 9 (5.75,10.5) | 7 (5,10) | 7 (4.5,10) |
| Abnormal brain MRI at onset, n (%) | 6/7 (85.7) | 54/59 (91.5) | 46/56 (82.1) |
| Relapse, n (%) | 71.4%（5/7） | 30.1%（19/63） | 19.3%（11/57） |
| FU time, median (IQR), mo | 26 (22.5,32.5) | 16 (13,21) | 19 (13,28) |

Abbreviations: Ab, antibodies; ADEM, acute disseminated encephalomyelitis; AQP4, aquaporin -4; FU, follow-up; IQR, interquartile range; MOG, myelin oligodendrocyte glycoprotein; OCB oligoclonal bands.
